# Motor Control Training for the Shoulder with Smart Garments

**DOI:** 10.3390/s17071687

**Published:** 2017-07-22

**Authors:** Qi Wang, Liesbet De Baets, Annick Timmermans, Wei Chen, Luca Giacolini, Thomas Matheve, Panos Markopoulos

**Affiliations:** 1 Department of Industrial Design, Eindhoven University of Technology, 5612 AZ Eindhoven, The Netherlands; luca.giacolini.16@ucl.ac.uk (L.G.); p.markopoulos@tue.nl (P.M.); 2 BIOMED REVAL Rehabilitation Research Institute, Faculty of Medicine and Life Sciences, Hasselt University, B-3590 Diepenbeek, Belgium; liesbet.debaets@uhasselt.be (L.D.B.); annick.timmermans@uhasselt.be (A.T.); thomas.matheve@uhasselt.be (T.M.); 3 Center for Intelligent Medical Electronics, Department of Electronic Engineering, School of Information Science and Technology, Fudan University, Shanghai 200433, China; 4 Shanghai Key Laboratory of Medical Imaging Computing and Computer Assisted Intervention, Shanghai 200000, China

**Keywords:** wearable system, rehabilitation, posture monitoring, compensatory movement, shoulder training

## Abstract

Wearable technologies for posture monitoring and posture correction are emerging as a way to support and enhance physical therapy treatment, e.g., for motor control training in neurological disorders or for treating musculoskeletal disorders, such as shoulder, neck, or lower back pain. Among the various technological options for posture monitoring, wearable systems offer potential advantages regarding mobility, use in different contexts and sustained tracking in daily life. We describe the design of a smart garment named *Zishi* to monitor compensatory movements and evaluate its applicability for shoulder motor control training in a clinical setting. Five physiotherapists and eight patients with musculoskeletal shoulder pain participated in the study. The attitudes of patients and therapists towards the system were measured using standardized survey instruments. The results indicate that patients and their therapists consider *Zishi* a credible aid for rehabilitation and patients expect it will help towards their recovery. The system was perceived as highly usable and patients were motivated to train with the system. Future research efforts on the improvement of the customization of feedback location and modality, and on the evaluation of *Zishi* as support for motor learning in shoulder patients, should be made.

## 1. Introduction

Shoulder problems are very common. In musculoskeletal rehabilitation, shoulder dysfunctions are the third most common complaint [1,2,3] and in neurological rehabilitation after stroke, shoulder pain affects one-third of stroke patients [4]. Shoulder problems affect functional arm recovery and functional arm use, which decreases daily life performance and autonomy.

**Figure 1:**
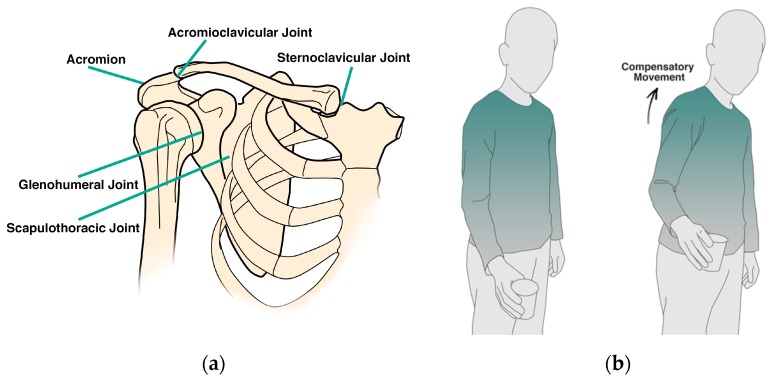
(**a**) Schematic anatomy of shoulder complex; (**b**) The compensatory movement in the task execution.

The complex movement of the shoulder (see Figure 1a) involves a combination of movements in the scapulothoracic, acromioclavicular, sternoclavicular, and glenohumeral joint. Alterations in trunk and scapulothoracic posture and movements, and in scapulothoracic muscle timing are related to the development of shoulder pain and dysfunctions, both in musculoskeletal and neurological (i.e., stroke) patients [5,6,7,8]. In reaction to pain or in order to unload painful structures, patients tend to develop altered movement patterns which can be seen as compensatory movements [9]. For example, in the performance of reaching tasks, patients with shoulder pain at the level of the glenohumeral joint, might develop aberrant movement patterns at the level of the scapulothoracic joint, in order to compensate for limited glenohumeral motion (see Figure 1b). This can be seen in the form of increased scapular elevation in the frontal plane or increased trunk lateral flexion. Compensations might also occur in the sagittal plane, i.e., scapular protraction or trunk flexion [10]. While compensatory movement helps patients to achieve their tasks, it can also obstruct the recovery progress and induce new orthopedic problems. In light of this, detecting and preventing compensatory movements warrants particular consideration for technology supported physical therapy treatment.

Research already indicates that in persons with musculoskeletal shoulder pain, a scapulothoracic posture retraining program results in reduced shoulder disability and pain, and ameliorated scapulothoracic movement and muscle activation patterns [11]. Thus, rehabilitation should involve correct positioning of the scapulothoracic joint by means of active muscle recruitment (motor control training) and re-education. Patients need to learn an optimal scapular orientation in relation to the thorax. Currently, patients learn such a ‘scapular setting’ by relying on verbal and auditory assistance of their therapist [12].

Posture monitoring technology can help detect and subsequently reduce the compensatory movement patterns of the scapula on the thorax in several ways. First, it can support the patient in executing scapular setting exercises by providing objective feedback on the scapular setting and trunk position which can contribute to the effectiveness of the training. Secondly, it can be supportive for therapists, since it can be an addition to the manual/verbal/auditory assistance they provide to patients. Lastly, it provides continuous and objective feedback, which can potentially improve the quality of the training. However, before a technical measurement or rehabilitation tool can be clinically applied, information about the ease of use of the system [13] and attitudes by users towards this technology is required. For this reason, it is of major importance to assess the opinion of the system’s users, i.e., therapists and patients, regarding their acceptance of technology, the usability and credibility of the proposed technology, and their motivation to use it.

## 2. Technologies for Shoulder Posture Monitoring

In broad lines, there are five categories of methods for shoulder posture monitoring [14]: mechanical, optical motion recognition system, depth camera-based system, robot-based solutions and wearable sensor-based system. The present research is concerned primarily with the latter of these approaches. While optical solutions and off body tracking systems are arguably more mature and reliable than wearable systems, they constrain training scenarios in several ways: optical motion trackers require a large space to operate successfully and suffer from potential occlusions. Higher-end infrastructure-based sensing systems such as the Vicon Tracker (Vicon Motion Systems, Ltd., Oxford, UK) can be costly, are location bound and require substantial effort for their installation, which can hinder their wider availability and application. On the other hand, consumer level tracking technology such as the Wii^®^ remote (Nintendo, Kyoto, Japan), not originally designed for the purposes of rehabilitation, can be appropriated for this purpose, but require substantial effort to wear, fix bands and make adjustments on the patient’s body.

There have been quite a few studies which examine upper body posture monitoring systems using wearable sensor technologies. A recent systematic survey [14] of wearable movement and posture monitoring systems for the upper body published in the last 6 years found that only 6 out of 45 publications concentrated on monitoring compensatory movements, while most systems located sensors on limb segments or joints for the measurements of range of motion, amount of use or other body segments position. A previous study [15] proposed that providing feedback may help decrease the compensation use. Dunne et al. [16] proposed an interactive system for upper extremity rehabilitation in children with cerebral palsy which monitors trunk movements using accelerometers providing feedback through games played on a multi-touch display. Alankus et al. [17] concentrated on reducing trunk compensatory movement during training in stroke rehabilitation. Ploderer et al. [18]proposed a system named “ArmSleeve”, supporting occupational therapists in stroke rehabilitation, involving exercise and activities addressing the control of compensatory movement. Lorussi et al. [10] integrated a scapular strain sensor that can detect scapula movement with respect to the sternum and rib cage in their wearable textile platform. Beursgens et al. [19], developed a vest for monitoring patient posture using a single sensor while playing a serious game intended to support arm-hand rehabilitation after stroke; however this vest did not fit the patient body tightly and thus could not maintain the sensor placement during training. Lin et al. [20] developed a wearable instrumented vest for posture monitoring which received positive feedback from their test participants. One of the motivations for the present research is that a higher integration of sensing components into the garment can potentially address this problem. It is noted that wearable systems with bulky sensor units [21] or sensors placed on the arm [10] are not able to track the scapular motion properly. So far little effort has been invested in establishing the usability of such systems.

A recent trend in this field pertains to the use of e-Textiles that integrate electronics for sensing and computation in clothing as a way to enhance aspects such as flexibility, comfort and aesthetic appeal. The integration of electronic components into fabric can be classified into three levels [22]: (1) Attached, (e.g., an accelerometer attached on a vest [16]); (2) Embedded, where electronic components are incorporated or embedded in the fabric, (e.g., wired components by conductive yarns and textiles [23]); (3) Integrated, were smart materials are knitted or woven into the garment’s fabric, (e.g., stretch-sensing fabric acting as a motion sensor [24]). The aforementioned survey [14] of wearable systems supporting upper body posture and motion monitoring shows that most systems can be categorized in the first level (attached), while the number of studies within the embedded level is growing as such systems hold the promise of proper fit, comfort and unobtrusiveness. There is yet little work in the third and integrated level; for example, a recent literature survey [25] about e-textiles in clinical rehabilitation only returned 11 studies for movement and posture.

While related research has been primarily concerned with demonstrating the required accuracy and clinical validity of measurements, less attention has gone towards integrating posture monitoring with motor control training applications, and towards their usability and aesthetics, and more general factors that are key to the eventual acceptance of this technology for patients, such as comfort and good wearability [26]. The development of the *Zishi* garment reported in this paper aims to address these abovementioned requirements. This article concerns the design and development of a wearable system to support motor control training. We first describe the development of a smart garment, which we call *Zishi*, to support trunk and scapulothoracic motor control training by monitoring compensatory movement and synchronously providing feedback. Then we outline how *Zishi* is used for scapula setting exercise training. Finally, the system is evaluated regarding ‘credibility and expectancy’, ‘usability’, ‘technology acceptance’ and ‘motivational aspects related to the use of the system’ by patients with musculoskeletal shoulder pain and physical therapists.

## 3. System Design and Development

### 3.1. Requirements

*Zishi* was developed in an iterative user-centered design approach; earlier iterations are reported in [27,28,29] to address the following requirements: (1) High accuracy in posture monitoring can be achieved by ensuring appropriate placement of sensors on the garment and tight fit of the garment to the body; (2) the garment should support reliable and customizable identification of inappropriate posture; (3) high wearability for the garment should involve comfort while wearing it, no constraint of body movement, a close fit but should be easy to put on and take off; (4) the system should provide real-time and easy to understand feedback. At least the design should make the device inconspicuous and resembling normal clothing rather than a medical device. The discussion below discusses these requirements specifically for monitoring the shoulder posture.

### 3.2. Calculation Methods

**Figure 2:**
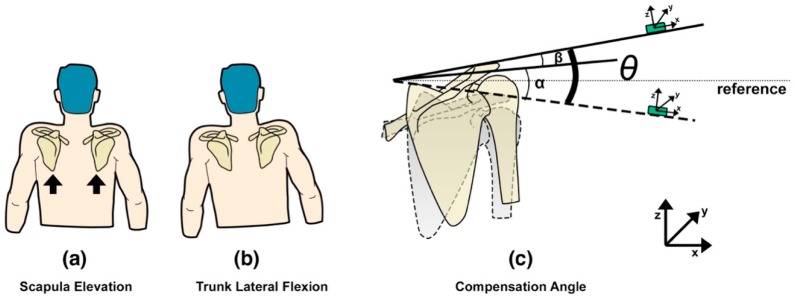
Calibration model of the compensatory movement from shoulder girdle.

*Zishi* has been designed to monitor and help prevent two kinds of compensation movements, in the frontal and sagittal plane, which can occur at the shoulder girdle and trunk, to avoid movement at the glenohumeral joint. Compensatory movement of the shoulder girdle is defined as the vertical displacement of the acromion sensor compared to the global coordinate. During rehabilitation exercises with arm movements below 60° in the sagittal plane, the scapula should perform a setting, which means that no excessive elevation or depression is allowed (see Figure 2a). Scapular elevation means that the scapula slides superiorly on the thorax, as in shrugging of the shoulders (moving the superior border of the scapula and the acromion in an upward direction). The acromion is the most lateral point of the shoulder girdle and its flat part is a suitable place to put a sensor on to identify whether the shoulder girdle is in an elevated or depressed position following the ISB (The International Society of Biomechanics) recommendations [30]. At the same time, patients may also develop a slight trunk lateral flexion leading to a lean of scapula (see Figure 2b). Figure 2c illustrates the compensation angle θ, consisting of α and β, which register the vertical deviation of the acromion compared to the neutral position because of the scapula elevation and trunk lateral flexion. The calculation takes as a reference the global coordinate sensor readings in respect to the frontal plane. The acromion sensor orientation is around the global Y, described using Euler angle following the Z-Y-X sequence (known as: yaw, pitch and roll).

**Figure 3:**
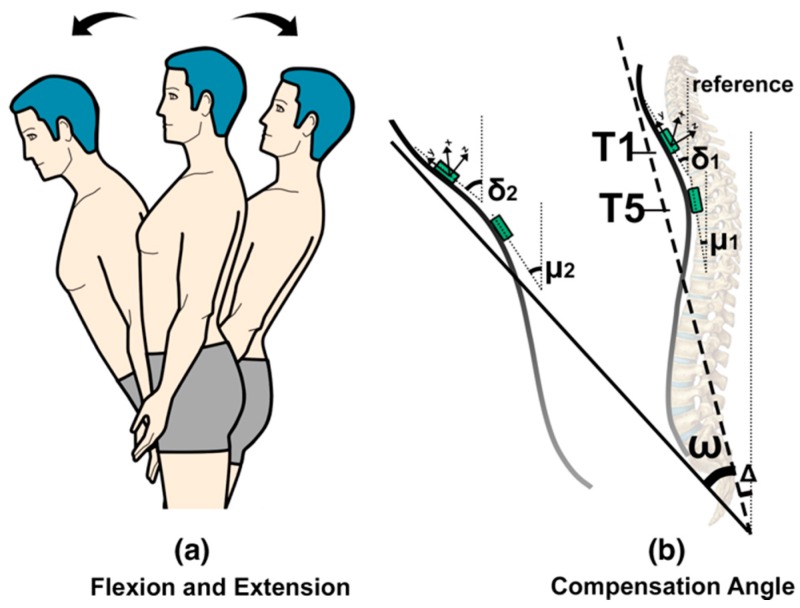
Calibration model of flexion and extension movement of torso.

**Figure 4:**
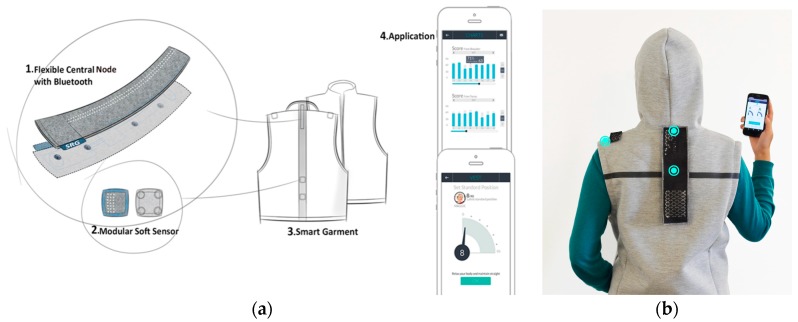
System concept overview of *Zishi*: (**a**) Concept illustration; (**b**) User wearing the garment, blue dots show the sensor positions.

Compensatory movement from the trunk is defined as the additional trunk flexion (anterior displacement), shown in Figure 3a. Two FLORA 9-DOF IMU sensors (Adafruit, New York, NY, USA) are used to evaluate the trunk posture in terms of flexion/extension accurately. The two sensors are positioned on the spine (C7/T1 and T4/T5) (Figure 3b). Sensor locations were based on the evaluation of the initial versions of the Smart Rehabilitation Garment system (reported in [28]). For detecting compensatory trunk movements in the flexion/extension direction, the average of the angles of the sensors with respect to the sagittal plane is calculated, which provides an indication of the upper thoracic angle (see Figure 4b). When the angles obtained from the IMUs (Inertial Measurement Unit) are δ and μ, then the average of δ and μ represents the estimated thoracic neutral angle Δ, and the compensation angle is the thoracic flexion angle ω, estimated as the average variance of δ and μ. Trunk sensor readings with respect to the sagittal plane are described using Euler angle sequence of rotation around the global axis X.

### 3.3. Zishi Concept and System Overview

*Zishi* can monitor compensatory movement from different parts of the upper body (shoulder, upper thoracic spine) and can be adjusted to fit the appropriate parts of the body. The garment design is shown in Figure 4a, featuring four parts: (1) a flexible central node equipped with two sensors, data processing components and communication module; (2) a modular soft sensor unit embedded in the strip; (3) a garment integrated with wearable smart textiles and connection points; (4) an application that runs on a handheld device. Figure 4b shows the sensor position on the torso and shoulder.

*Zishi* is designed for use during rehabilitation training to support scapular motor control training. it can be used in different populations and for a variety of patient groups whose arm function or shoulder pain might improve from such scapular motor control training. In this paper, we focus on the application for patients with musculoskeletal shoulder pathologies. For this group, *Zishi* provides real-time tracking feedback of the trunk and shoulder (including scapular) posture and movement in order to: (1) improve patients’ posture awareness to motivate and help rehabilitation training; (2) provide postural feedback supporting motor learning; (3) support independent training at a rehabilitation center or at home.

### 3.4. Garment Design and Conductive Textile Integration

**Figure 5:**
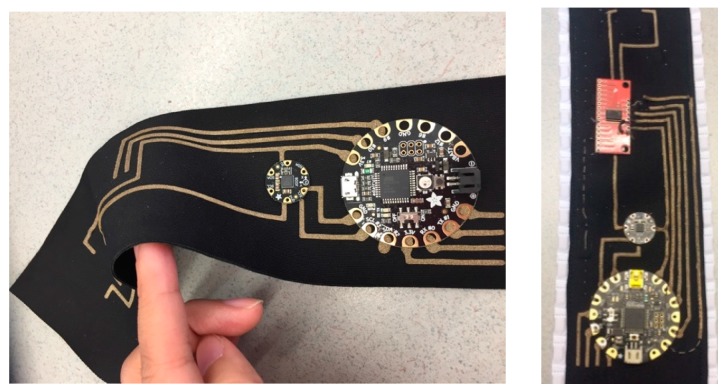
Conductive textile-based flexible traces.

The flexible central node consists of the following components: (1) Two IMU sensors; (2) a multiplexer board to support multiple sensors in the system; (3) a Bluetooth module for wireless communication between the microcontroller and smart device; (4) FLORA, an Arduino-compatible microcontroller to which all other components are connected to; (5) 3V lithium battery. A laser-cut conductive textile pattern was applied as the flexible traces to connect the electronic components (shown in Figure 5).

**Figure 6:**
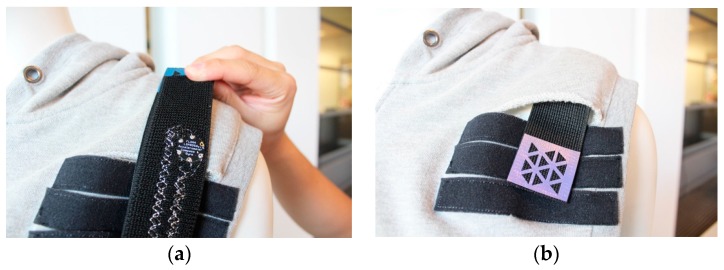
Garment Implementation: (**a**) Sensor embedded in a Velcro strap by coated conductive yarn; (**b**) Velcro adjustments.

Addressing the proposed design criteria, a dedicated garment design is essential to integrate all the parts in the systems. Instead of the earlier 2-piece design [27,28] in which the different garment parts were attached to each other with a Velcro strip, we opted for a zipped vest of soft material, which is easier for patients to put on and take off. In order to guarantee a precise sensor placement over time in the pre-defined position, the sensor is flat (height 0.8 mm, diameter is 16 mm) and sewn by coated conductive yarn on a soft elastic strap (Figure 6a) with a Velcro fastened at its end. One side of the strip was fixed on the garment while the other side was flexible for adjustment (Figure 6b). Thanks to the adjustable design, the sensor could be located at the flat part of acromion. The movement of the acromion during the exercises is very limited, i.e., the exercises’ aim is to keep the scapulothoracic joint still (setting phase—scapulothoracic motor control exercise) while the arm is elevated (in the limited range of motion that is possible without scapulothoracic movement). In this way, the accuracy was ensured by the sensor position and the fit design of the garment (in different size and Velcro fix). This allows to adjust the sensor placement to people with different body sizes.

Aiming for an unobtrusive and comfortable garment, a conductive network was made using coated conductive yarns sewn on the garment, which served as a fabric-friendly embedded platform for the sewable electronic components described above. In this way, the garment looks like daily clothing in order to improve user acceptance, see Figure 4b.

### 3.5. Feedback Strategy

**Figure 7:**
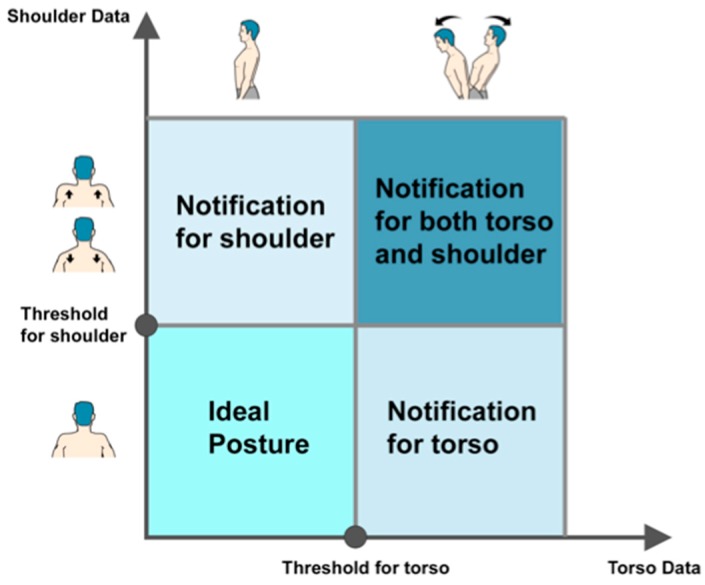
Feedback strategy.

Feedback is important during rehabilitation training, for speeding up learning processes, for augmenting treatment effects and sustaining motivation [31]. *Zishi* provides both Knowledge of results and Knowledge of performance feedback [15], the latter consisting of real-time kinematic feedback (e.g., shoulder girdle elevation value) and notifications of posture deviations beyond a preset threshold. *Zishi* provides continuous feedback by means of visual and auditory information through a connected handheld device (smartphone or tablet), and vibration notifications delivered through the garment. The system is able to notify patients when the readings of the sensors exceed their personalized bandwidth. The sampling frequency of the sensor system is 50 Hz, while the frequency of the visual display (pointer rotation) was set at 30 Hz because of the data synchronization with the smartphone to ensure a smoothly visual display. During the experiment, subjects performed the tasks in a controlled manner (i.e., no explosive movements), similar to their daily training. In this way, the system is capable of monitoring the posture in real-time and providing subsequent feedback with inconspicuous delay. A therapist can calibrate the device to the patient by identifying a neutral shoulder girdle and trunk postures for this patient and, depending on the goals and progress of training, set a personalized training bandwidth around this position that corresponds to the allowed compensation range. The neutral position (the position in which we calibrate) is the midstance between protraction and retraction, and the midstance between elevation and depression. This procedure works for patients in different pathologies and health conditions. Figure 7 illustrates the feedback strategy overview. Contrary to posture monitoring devices that are currently available, supporting consumer technologies, setting personal thresholds is an important step that should ideally be carried out together with the therapist.

**Figure 8:**
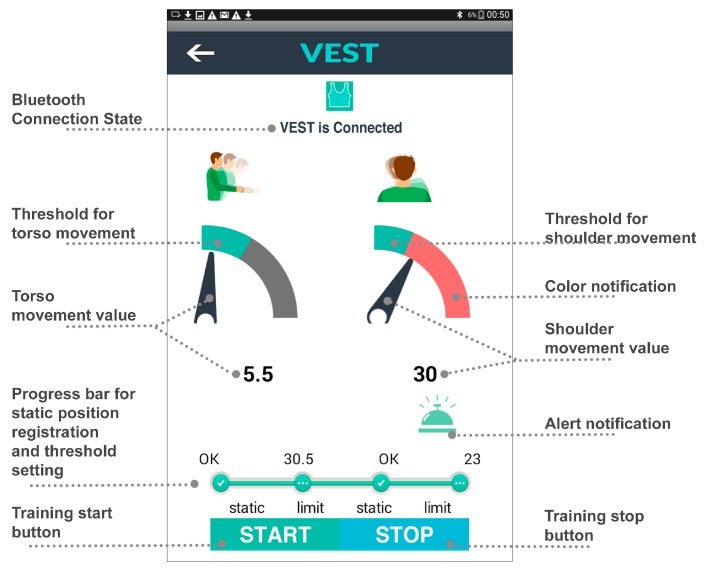
Interface Design.

**Figure 9:**
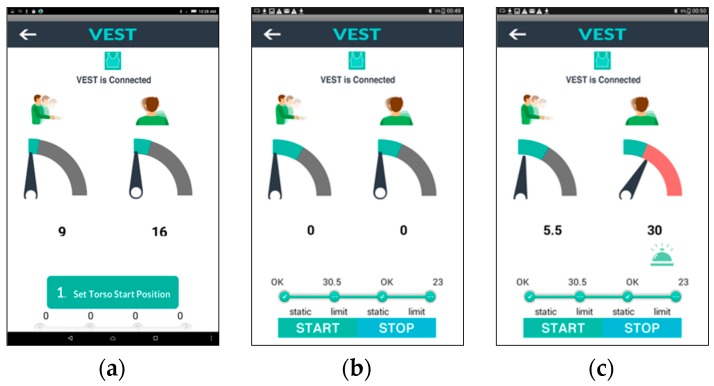
Interfaces design in different stages. (**a**) Automatically connected. (**b**) Set personalized value of start position and threshold. (**c**) Visual feedback when the shoulder value is over range.

Figure 8 illustrates an overview of the elements in the user interface and Figure 9 shows the interface during the training execution. The workflow of the application operation is as follows:

During the first stage (a) the user starts the system which pairs the App to the garment automatically. The user’s movements are visualized by a rotating pointer and a numerical reading, presenting the movement angle for flexion/extension of torso and the elevation/depression of shoulder.

In the subsequent stage (b) the device is calibrated, as discussed already, by setting the neutral position and training bandwidth. Ideally, calibration should be performed with the therapist. Patients may follow instructions to sit in a neutral posture and keep their upper body static to register the “0” point of the scale as the neutral position of torso and shoulder separately. Then the patient is asked to bend forward until the App displays the target threshold that the therapist indicates as the maximum acceptable; this process is repeated for setting shoulder elevation threshold while shrugging the impaired shoulder. The progress bar visualizes the current phase of static position and threshold setting of torso or shoulder.

Figure 9c shows the interface during the actual training after the “start” button has been pressed. The green region in the dial shows the intended training bandwidth area. Patients can watch the screen while training and can see whether they compensate excessively and perhaps try to adjust their movement not to exceed the allowed range. The remaining grey part of the dial will turn red if the pointer exceeds the threshold. At the same time, an alarm icon will appear for 2 s with an audio alert and vibration notifications on the corresponding part of the garment. In this way, users can be made aware of their compensatory movements and learn to control them. The customized threshold setting serves as a reference target for each motion cycle. Providing real-time feedback, we aim to support correct movement execution and enhance motor learning, and thereby training effectiveness.

## 4. Evaluating Patient Attitudes towards the System

The accuracy of the system has been evaluated [28] by comparing the system to a commercial optical tracker that uses infrared cameras to track optical markers. The average root-mean-squared error (RMSE) between the angle estimation was 3.57° during the tasks from 15° to 75° among seven subjects. As such, the system achieved comparable accuracy to the current state of the art in wearable sensors for rehabilitation [32]. The aim of this study is to evaluate attitudes regarding the usability, credibility, acceptance, and motivational aspects of technology-supported postural feedback during scapular training in patients with musculoskeletal shoulder pain and in physical therapists who treat patients with shoulder disorders. The study received ethics clearance from the ethics boards of Jessa Hospital (Hasselt, Belgium) and Hasselt University. The study comprised of two parts; in the first part, patients were asked to use *Zishi* while executing scapular setting exercises and in the second part, physical therapists tried out *Zishi* themselves and evaluated the system as a therapy aid.

### 4.1. Participants

Eight patients with musculoskeletal shoulder pain receiving rehabilitation training and five physiotherapists from the rehabilitation center of Jessa Hospital were recruited and signed the informed consent before the study. Eight patients with pain from musculoskeletal origin (five females and three males) agreed to participate in our study. Their ages ranged from 45 to 59 years (M = 50, SD = 6.44) and they had been following shoulder rehabilitation training for 9.7 months on average (SD = 5.8). Their mean SPADI score was 45.3 (SD = 15.3%).

The inclusion criteria for the shoulder patients were: (1) main complaints at the shoulder girdle; (2) older than 18 years of age; (3) presence of at least one of the following signs: positive Neer test, positive Hawkins-Kennedy impingement, painful arc during active abduction/flexion, pain during one or more of following movements: external rotation/internal rotation/ abduction/ flexion; (4) understanding of spoken and written Dutch.

The exclusion criteria were: (1) Surgery at the shoulder complex or cervical spine in the last 6 weeks, (2) comorbidity: paresis and sensory problems of neurological origin/diabetes mellitus/ rheumatoid arthritis, pain severity of 8/10 or more in the last 48 h, adhesive capsulitis/frozen shoulder, (3) having any insurance compensation claims in progress.

### 4.2. Materials

*Zishi* was made available in two sizes. The materials for the experiment also included an Android-based tablet with the App installed, an adjustable shelf, a cooking pot (weight 300 g, size 6 inches) and a bottle of water (0.5 L). Although *Zishi* is capable of providing feedback in three different modalities, only visual and audio channels were enabled during the experiment to prevent information overload (vibrotactile feedback was disabled).

### 4.3. Protocol

**Figure 10:**
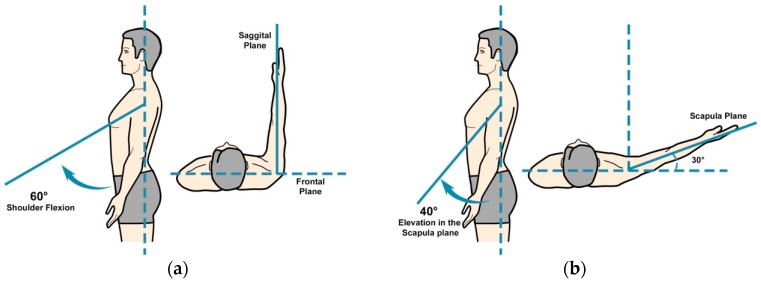
Movement description: (**a**) Shoulder flexion; (**b**) Elevation in scapula plane.

**Figure 11:**
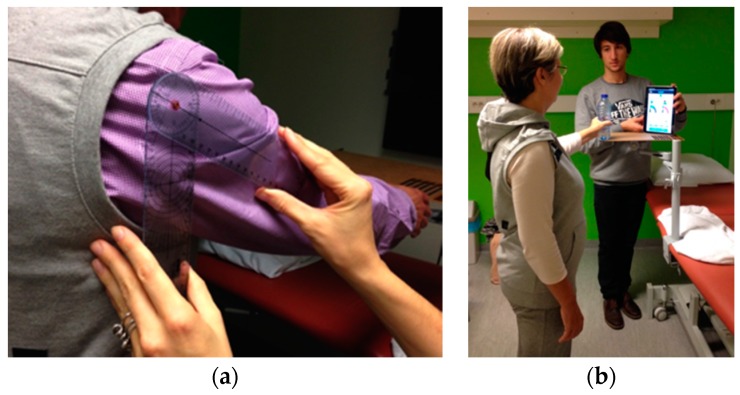
Task Execution: (**a**) Standardized calibration of arm movement with goniometer; (**b**) The subject is performing task 4, lifting the bottle to the board.

Based on discussions with therapists, it was decided to only focus on the right part (shoulder girdle feedback) of the interface to avoid concurrent feedback of two different aspects of posture (i.e., from the trunk and the shoulder girdle). It was assumed that focusing on two aspects of posture would be too difficult for patients given that they were not yet familiar with the system and its feedback. Before starting the experiment each participant filled in two pre-test questionnaires: a socio-demographic questionnaire (name, date of birth, gender, height, weight, contact information, shoulder pain suffering time and position, handedness and whether they had surgery on shoulder) and the Shoulder Pain and Disability Index (SPADI) questionnaire [33], a self-administered inventory to gauge the shoulder pain they experience at the moment and the disability of shoulder functioning. The SPADI questionnaire generates a score ranging between 0 and 100, reflecting the amount of disability of a person, with higher scores corresponding to a higher degree of disability. Researchers, who are also musculoskeletal physiotherapists, demonstrated *Zishi* to the participants and explained its operation and interface contents. Subsequently, the participant put on the garment and ran through the calibration procedure. All tasks were performed in a standing position, with help from a researcher when necessary. During task execution, the patient was instructed to stabilize the scapula on the thorax and avoid inappropriate scapular elevation or depression. The neutral scapular position was calibrated, and the threshold for allowed compensatory movement was set at 10° of scapular elevation or depression. Test participants were asked to perform the following tasks:

(1) Task 1: analytical shoulder flexion. The subject was asked to perform 60° of shoulder flexion with the thumb up and the elbow extended (see Figure 10a). A bar was placed in front of the patient at 60° to indicate the appropriate level of flexion. The range was determined with a goniometer, as shown in Figure 11a.
(2) Task 2: functional shoulder flexion, placing a cooking pot. The subject was asked to place a cooking pot from a plate on a shelf that was located in front of him/her. The height of the shelf and the distance from the patient were standardized. The subject started with his arms alongside his body and with the elbows in 70° of flexion (determined with goniometry). The height of the shelf was adjusted accordingly. The patient had to perform 60° of shoulder flexion with extended arms, to place the pot on the shelf. Once subjects had placed the cooking pot on the shelf, they were asked to put it back on the shelf in front of them.
(3) Task 3: analytical elevation in the scapular plane. The subject was asked to perform 40° of shoulder elevation in the scapular plane (30° in front of the frontal plane) with an extended elbow and with the thumb pointing upward (see Figure 10b). A bar was placed in the scapular plane, next to the patient at 40° of humerothoracic elevation to indicate the appropriate level of elevation. The range was determined with a goniometer.
(4) Task 4: functional elevation in the scapular plane. The patient was asked to place a bottle of water (0.5 L) on a shelf that was located next to him in the scapular plane. The height of the shelf and the distance from the patient was standardized. At the starting position, the patient had his arms alongside his body and his elbows in 70° of flexion (measured with a goniometer). The bottle was in the hand of the tested arm side. The shelf was placed so that the patient had to perform 40° of scapular plane shoulder elevation with an extended arm to place the bottle on the shelf. Figure 11b shows a subject performing task 4.

Five therapists participated in the second part of the study. They performed the same protocol as described above, in order to gain a first-hand experience of the system before providing their own appraisal of it.

### 4.4. Outcome Measures

At the end of the test sessions, participants were asked to fill in a number of questionnaires that assessed different aspects of the system.

#### 4.4.1. Credibility and Expectancy

To evaluate whether participants think that *Zishi* is a potentially credible aid for treating shoulder pain (credibility), and whether they feel it will facilitate the improvement of their condition (expectancy), we asked them to complete the Credibility Expectancy Questionnaire (CEQ [34]). The CEQ includes a credibility factor (to indicate how believable, convincing and logical treatment is) as well as an expectancy factor (expected improvements). In therapists, only the questions related to credibility were asked, as they are not assumed to have a musculoskeletal problem they are treating with the device. The questionnaire consists of four questions on what subjects ‘think’ and two questions on what subjects ‘feel’, while the factor credibility is derived from the first three thinks questions and factor expectancy is derived from the remaining questions.

#### 4.4.2. Intrinsic Motivation

As we assume that interactivity will make exercise more engaging and will increase patients’ motivation to train, we asked them to fill in the Intrinsic Motivation Inventory (IMI [35]). The full version of IMI consists of 45 questions addressing seven subscales. Since the subscale ‘Perceived Choice’ is not relevant for our system, we only focused on the other 6 subscales including ‘interest/enjoyment’, ‘perceived competence’, ‘effort/importance’, ‘value/usefulness’, ‘relatedness’ and ‘pressure/tension’. The ‘interest/enjoyment’ subscale is considered as the self-report measure of intrinsic motivation. The ‘perceived competence’ subscale shows how capable the subjects feel and theorized as predictors of intrinsic motivation. The ‘effort/Importance’ and ‘pressure/tension’ subscales respectively measure a subject’s effort investment and pressure during task performance. The subscale ‘value/usefulness’ aims to capture the extent to which people internalize and develop more self-regulatory activities when experience is considered as valuable and useful for them [36]. Questions in the subscale ‘relatedness’ are designed to reflect the degree of participants’ perceptions and expectations of social connection when using the system.

#### 4.4.3. Technology Acceptance and Usability

To evaluate whether participants would be likely to use such a device, we assessed technology acceptance using ‘Unified Theory of Acceptance and Use of Technology’ (UTAUT [37]). Developed in the field of information systems management, the UTAUT inventory measures a variety of factors that are known to predict use of technology. However, UTAUT is not very explicit about system usability, which is an important concern for interaction design. For this reason, participants were also asked to complete the Computer System Usability Questionnaire (CSUQ [38]), developed by IBM, which is a short, reliable, and widely used questionnaire for assessing system’s usability. The CSUQ questionnaire consists of 19 items for measuring user satisfaction with four perceptions of satisfaction: overall satisfaction (Q1–Q19), system usefulness (Q1–Q8), interface quality (Q16–Q18) and information quality (Q9–Q15). Finally, we also asked several questions addressing the general experience and quick impression of the system’s usability: two rating questions (R1—“How easy can you understand the feedback?”; R2—“How easy was it to take the vest on and off?”) and two open questions (O1—“What do you like about the system?”; O2—“What would you change about the system?”).

#### 4.4.4. Credibility and Expectancy for Therapists

The therapists filled in the credibility component only of the CEQ questionnaire. Furthermore, to assess the general impression and usability, and to receive suggestions for improvement of the interface design, several open questions were asked (for example, “What do you think about the arrow—was it clear? Should you replace it by an avatar? Or by an image of yourself?” To learn about therapists’ opinions about the feedback strategy, we asked the questions: “Do you prefer feedback during or after the exercise?” and “Do you prefer feedback about the manner of movement (knowledge of performance) or only on the result of movement (knowledge of results)?”.

## 5. Results

Table 1 presents the group Median, IQR scores and one-sample Wilcoxon signed rank test results of the different questionnaires and their subscales in comparison to the neutral score of each questionnaire.

### 5.1. Patients’ Evaluation

#### 5.1.1. Measurement Results of Credibility and Expectancy

Patients scored highly on both the credibility and the expectancy factor (see Table 1) as both scores were higher than the neutral score of 13.5 (credibility Median = 22.5, *p* = 0.011, IQR = 3.5; expectancy Median = 20.2, IQR = 3.55; *p* = 0.012).

#### 5.1.2. Measurement Results of Intrinsic Motivation

**Figure 12:**
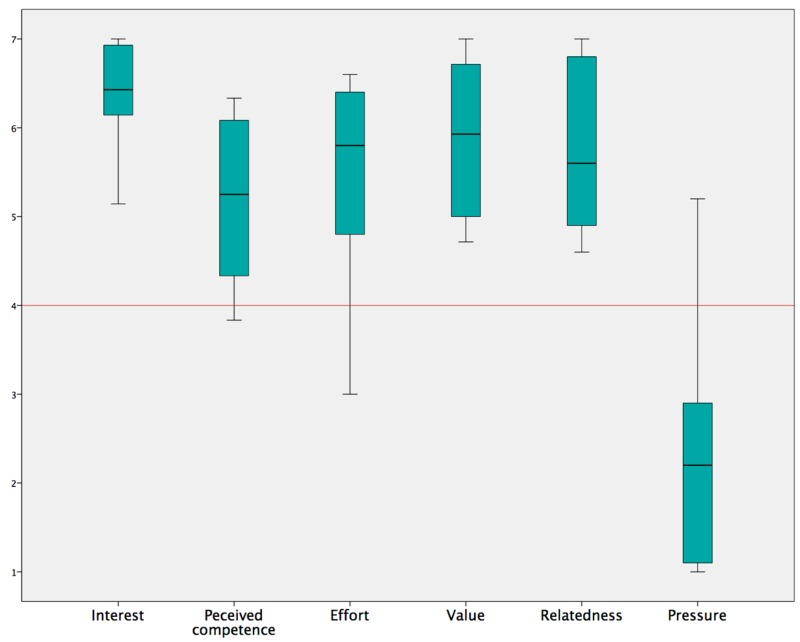
Subscale findings of the Intrinsic Motivation Inventory questionnaire evaluated in patients with shoulder pain.

Figure 12 illustrates the scores given for the six subscales of the IMI questionnaire. For all subscales, patients scored well above the neutral score of 4 (see also Table 1), with the exception of the subscale ‘pressure/tension’ which is a negative predictor (“less pressure is better”) and should be ideally below the neutral. The ‘interest/enjoyment’ subscale, which is considered the most direct self-report measure of intrinsic motivation, indicated that the subjects were significantly more than neutral motivated to use the system (median = 6.43, IQR = 0.82; *p* = 0.012). The ‘perceived competence’ and ‘effort/importance’ subscales resulted in acceptable scores and the ‘value/usefulness’ and ‘relatedness’ subscales were scored highly. The low score of subscale ‘pressure/tension’ showed that subjects did not experience pressure or tension during the task.

#### 5.1.3. Technology Acceptance and Usability

**Figure 13:**
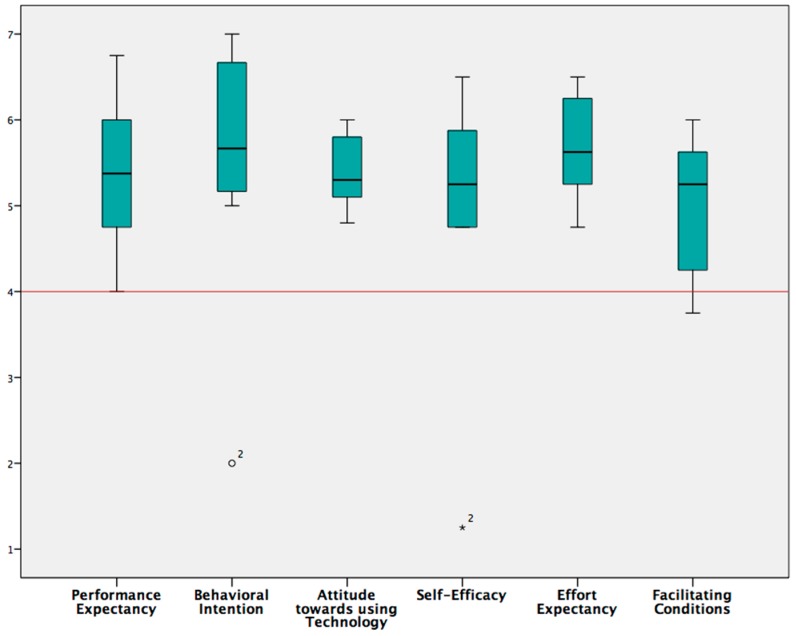
Technology acceptance was measured with the UTAUT questionnaire, achieving positive evaluations by the participants.

We used UTAUT (Unified Theory of Acceptance and Use of Technology) to assess the constructs that influence technology acceptance (Behavioral intention) and use (Behavior) of the *Zishi* system. The results (see Figure 13) indicate that the subjects believed that *Zishi* may help them (Performance expectancy) and that they expect that it will require little effort to use (Effort expectancy).

In general, patients were positive towards using the *Zishi* for training their shoulder, reporting high scores on the behavioral intent scales (Behavioral intention). Patients further moderated contextual factors that would influence their ability to use the *Zishi* positively (Facilitating conditions) which suggests that they believe that the technology could be integrated in the current treatment practice. While an initial analysis showed that behavioral intention and self-efficacy were not significantly higher than neutral, we noticed that one subject was using the lowest possible score on these scales. This could suggest that the interface needs improvement or that perhaps self-efficacy would improve with practice. However, as this score was in extreme contrast to the other scores of that patient and the scores by other patients, we suspect that the patient might have misunderstood the question. As this score is an outlier, removing it from the analysis, it is shown that scores are significantly higher than neutral for both behavioral intention and self-efficacy (see Table 1, *p* = 0.018 for both subscales). Table 1 shows that system usefulness and interface quality were both rated very high. Overall, the results indicate the high appreciation and good usability since the questionnaire comprises questions on the ease of use, ease of task completion, ease to understand and learn, and interface comprehension. However, three subjects found it difficult to understand and answer items Q11, Q12 and Q15. As a result, we did not report subscale information quality and overall satisfaction. However, it is noteworthy that (Q13—“*The information provided by the system is easy can you understand the feedback*”) was rated positively (Median = 10, IQR = 1.75, on a scale of 10).

In addition, the garment was found easy to take on and off (Median = 10, IQR = 1.75). Regarding to the first open question (O1—“what do you like about the system”), only five subjects responded. They said that *Zishi* is good for their training because of the correct, direct and understandable feedback. One subject even mentioned that she would recommend it to other shoulder pain patients, while two subjects mentioned that the app did not seem easy to use at home for older people. Only one subject replied to the second open question (O2—“what would you change about the system”) requesting to make the pointer sharper.

### 5.2. Therapists’ Attitudes

The five therapists gave credibility accreditation (Median = 20, IQR= 5.5), which indicates that therapists find the system credible for shoulder pain rehabilitation. Concerning the open questions, three therapists agreed with the current design, while two therapists proposed that replacing the arrow by an avatar would increase motivation. All therapists indicated that providing concurrent feedback about the movement is essential. Three therapists stated that both concurrent feedback and end of session feedback would be appropriate. On the question “How do you want patients to receive feedback?”, all therapists missed a summary of the performance progression and would like a control to choose which feedback is shown to the patient (torso or shoulder). The therapists complained about having to hold the tablet during training requiring a stand on which to place the tablet during training. The positive aspects were summarized as follows: “Useful, user-friendly, easy and no preparation time, can motivate patients.”

## 6. Discussion and Future Work

Evaluation of patient attitudes towards the prototype revealed a very positive perception of the system concept. *Zishi* is perceived as credible and usable, while training with the system is overall experienced as motivating. It is promising that some participants expressed a wish to take the garment home for more exercises. *Zishi* appears to strike a balance between accuracy and comfort, it is easy to use and does not require a lot of space. Compared to wearable sensors that have to be attached to the body with straps or adhesives (e.g., the XSens system), designing the wearable to look like everyday clothing can make it unobtrusive, especially if extra attention is paid to aesthetic/fashion aspects. The wearability of the system enables training daily living tasks as has been demonstrated by the tasks included in the evaluation of our experiment (e.g., placing the cooking pot on a shelf). This ensures the relevance of training to improving the daily life of patients but also the potential to extend scapular setting training at home.

The gauge in the *Zishi* interface accompanied by a numeric reading is clear and efficient for simple training tasks. Although it is valuable to monitor compensatory movement from the trunk and shoulder at the same time, the combined dials pose a large cognitive load to patients and for this reason we did not expose participants to both dials at a time, especially because there was not the opportunity for them to familiarize with the system through repeated training sessions.

Half of the participants mentioned their preference and trust in concurrent feedback, which is known to be effective for beginning users [39], since patients can correct the posture immediately. Based on the comments from the patients, it appears that the bandwidth feedback strategy has been useful for improving the user’s posture awareness. Audio feedback, triggered when participants moved out of the training bandwidth, is helpful without requiring visual attention during functional tasks.

By using conductive materials such as conductive yarn and textiles, *Zishi* supports a reconfigurable and robust connection between the garment and sensing package, ensuring both the wearability and aesthetic quality. The high intrinsic motivation of patients participating in the evaluation, and the positive credibility and acceptance scores indicate that patients are very positive about integrating *Zishi* into their current training. A larger scale study may also be able to explore in more depth potential effects of age, gender, experience and voluntariness of use on the attitudes regarding this technology.

Several limitations of our work are: (1) this study examined patient attitudes in a small scale study and with limited exposure to the system. As such, it cannot make claims regarding the effectiveness of the system to support training, for which larger sample sizes and training outcome measures would be needed; (2) The study reports positive attitudes towards this solution but does not compare against similar systems and traditional therapy.

Future research should: (1) Enhance the feedback design to allow for customization of content, modality and scheduling, and summary feedback. The interface will provide a checkbox so that beginners could start with only information from parameters which are trained (e.g., if a focus is toward correct shoulder posture, only feedback about shoulder will be made available). More rewarding elements towards their lower compensation movements will be added, for example increasing scores, happy face and encouraging word “well done”. In this way, the smart garment system provides feedback on the manner of task performance and will increase a person’s confidence in doing the task correctly, improving patients’ self-efficacy; (2) clinically evaluate the effectiveness of *Zishi* for scapular motor control training in comparison to training with feedback from a therapist in patients with scapular dyskinesis. Based on initial results, a sample size calculation will be performed; (3) include field tests for *Zishi* training in a home environment; if the system proves to be robust and usable enough to be used independently by patients at home, then the question arises whether *Zishi* could potentially increase the training time and training efficiency of the scapular setting exercises; (d) measure other exercises for which compensatory movement is expected (e.g., in the protraction direction during internal rotation of the arm). The shoulder complex and the cervical spine are closely linked, as are the thoracic and lumbar spine. It is of additional value to track all regions together as an adverse posture in one region influences the posture in other regions.

## 7. Conclusions

This paper argues that wearable posture and upper body movement monitoring technology can be of great value for rehabilitation training. A number of requirements for wearable rehabilitation garments have been identified. These requirements have been addressed in the iterative design and development of the *Zishi* system. This work illustrates how technology can monitor compensatory movements for supporting a shoulder-training program. The evaluation demonstrates the credibility of the approach, the high usability of the system, and its positive reception by patients and therapists in terms of technology acceptance and motivation to train with the system.

Now that we have knowledge of the feasibility of the system by the end-users in a clinical context, a trial on the clinical effectiveness of the system, in comparison to traditional rehabilitation methods, is the next imperative step. Future studies should examine whether the system is effective in motor learning for shoulder patients, and whether it helps achieve gains the quality and intensity of the rehabilitation. Further, the potential of the device to support independent rehabilitation training needs to be investigated in the future.

This paper contributes to the growing research literature regarding the use of wearable solutions for supporting rehabilitation training, with a design that has emphasized wearability, comfort and ease of use. We anticipate such integration of wearable sensing in clothing to provide several advantages over competing technological solutions and to provide great improvements on rehabilitation training quality and intensity.

## Figures and Tables

**Table 1:** Overview of system scores.

	Factor	Neutral	Median(IQR)	Sig
**Credibility/Expectancy (CEQ)**	Credibility	13.5	22.5 (3.5)	0.011
	Expectancy	13.5	20.2 (3.55)	0.012
**Intrinsic Motivation (IMI)**	Interest/Enjoyment	4	6.43 (0.82)	0.012
	Perceived competence	4	5.25 (1.96)	0.028
	Effort/Importance	4	5.8 (1.9)	0.025
	Value /Usefulness	4	5.93 (1.93)	0.012
	Relatedness	4	5.6 (2.05)	0.012
	Pressure/Tension	4	2.2 (2)	0.025
**Technology Acceptance (UTAUT)**	Performance expectancy	4	5.37 (1.75)	0.018
	Behavioral Intention	4	5.67 (1.58)	0.058
	Attitude towards technology	4	5.3 (0.85)	0.012
	Self-Efficacy	4	5.25 (1.19)	0.16
	Effort expectancy	4	5.62 (1)	0.011
	Facilitating conditions	4	5.25 (1.44)	0.024
**Usability (CSUQ)**	System usefulness	4	5.63 (1.53)	0.012
	Interface quality	4	5.67 (1.33)	0.011

Scores range from 1 to 7 for all factors apart from credibility and expectancy which range between 1 and 27. Abbreviations: IQR = Interquartile Range, Sig = Significance level of Wilcoxon Signed-Rank Test for One Sample, comparing to the neutral score of each scale.
